# Assessing the capacity for conflict and health research in Lebanon: a qualitative study

**DOI:** 10.1186/s13031-020-00304-x

**Published:** 2020-08-18

**Authors:** Nassim El Achi, Gladys Honein-Abouhaidar, Anthony Rizk, Elsa Kobeissi, Andreas Papamichail, Kristen Meagher, Abdulkarim Ekzayez, Ghassan S. Abu-Sittah, Preeti Patel

**Affiliations:** 1 R4HC-MENA, Conflict Medicine Program, Global Health Institute, American University of Beirut, Beirut, 1107 2020 Lebanon; 2 Hariri School of Nursing, Global Health Institute, American University of Beirut, Beirut, 1107 2020 Lebanon; 3 Conflict Medicine Program, Global Health Institute, American University of Beirut, Beirut, 1107 2020 Lebanon; 4 School of Politics & International Relations, Queen Mary University of London, London, E1 4NS UK; 5 R4HC-MENA, Conflict and Health Research Group, Department of War Studies, King’s College London, London, WC2R 2LS UK

**Keywords:** Capacity strengthening, Lebanon, Research, Health, MENA, Conflict, Needs assessment

## Abstract

**Background:**

Conflicts pose new challenges for health systems, requiring rapid and practical approaches to meet emerging needs on the ground. Lebanon has been highly influenced by surrounding conflicts in the Middle East and North Africa (MENA) region, especially the Syrian crisis. Strengthening research capacity to collect evidence on conflict in the MENA region and beyond is crucial to inform healthcare policy and practice. For targeted capacity strengthening interventions, the main objective of this paper is to present key findings of a needs assessment of conflict and health research in Lebanon. This will support recent efforts to scale up context-specific policies, interventions to strengthen the country’s health system, and research capacity.

**Methods:**

The study is based on 30 semi-structured interviews with key informants such as specialist academics, humanitarian workers and public sector officials.

**Results:**

Despite being ranked third in the number of publications on biomedical and health research per capita in MENA and in hosting reputable universities which are considered central academic hubs in the region, lack of nationwide research culture, insufficient funding and limited access to data were reported to be major challenges for health researchers in Lebanon. Even with the ongoing efforts, poor impact of research on policy continues to be a persistent gap. Large disparities in research capacities and taught skills were reported between different universities in Lebanon, with a disproportionate emphasis on quantitative over qualitative skills. Most medical students are not trained to conduct research or to practice in conflict settings. Concerns were also expressed regarding the ethics of research conducted, specifically by local non-governmental organizations.

**Recommendations:**

To conduct contextualized trainings on research skills with a stronger focus on qualitative approaches, medical practice, and ethical research in conflict. To better involve policymakers in designing research agendas by organizing multiple stakeholder meetings.

**Conclusion:**

The study indicates that health research in Lebanon is characterized by considerable strengths in terms of human capital and research capacities of certain universities. However, the Lebanese research infrastructure needs further development in terms of ensuring sustainable funding, providing access to data, teaching qualitative research skills, conducting ethical and multidisciplinary research, and promoting cross-sectoral knowledge transfer.

## Key messages

- Lebanon has been highly influenced by surrounding conflicts in the Middle East and North Africa (MENA) region, especially the Syrian crisis
- Lebanon is considered a major hub for health and conflict research in the region
- Lebanon has considerable health research strengths in terms of human capital and research capacities, yet there are significant disparities across Lebanese universities
- There is a need to provide context-specific trainings on health and conflict research skills with a stronger focus on qualitative approaches, medical practice, and ethical research
- Policymakers should be included in the early stages of setting health research priorities and research designs to promote evidence-based policies
- Lebanese health research infrastructure requires further development in terms of ensuring sustainable funding, providing access to health data, conducting ethical and multidisciplinary research, and promoting cross-sectoral knowledge transfer

## Introduction

Research capacity strengthening has been a top priority for the World Health Organisation (WHO) since 2003, paving the way for substantive investments from research funding agencies and international institutions [1]. While evidence generated from health research is crucial for addressing health and development challenges in countries and regions affected by armed conflict [2], the capacity to conduct locally relevant health research faces specific challenges in contexts affected by acute and protracted conflict. In conflict settings, health research is often deprioritized as focus shifts to improving security, conflict resolution, short-term humanitarian responses and managing forced migration [3]. However, the protracted nature of contemporary conflicts and their long-term impact on health provision has led to an increased demand and willingness to conduct and strengthen health research capacity in conflict-affected settings [4–6], including countries in the Middle East and North Africa (MENA) region [7].

The MENA region is no stranger to conflicts, many of which are complex and protracted (See Table 1 for definitions). The first wave of the Arab Spring in 2011 included large-scale mobilizations and political upheavals across the region, with armed conflicts developing in Libya, Yemen, and Syria and mass displacement leading to the worst humanitarian crisis since World War II [17]. In 2018, 37% of 70.8 million people displaced worldwide originated from the MENA region [18]. This process continues as more recently in 2019, the second wave of the Arab Spring emerged in Algeria, Sudan, Iraq and Lebanon, with anti-government protests installing transitional governments amid calls for major reforms [19].

**Table 1:** Key definitions

**LMIC (Low and Middle-Income Country):** According to the World Bank’s definitions, drawing on 2020 figures, low-income economies have a gross national income (GNI) per capita of $1025 or less; the GNI per capita of **lower middle-income** is between $1025 and $3995; and **upper middle-income economies** like Lebanon have a GNI per capita of between $3996 and $12,375 [8].	
**Capacity strengthening:** As a working definition, capacity strengthening can be understood as a process of developing, upgrading and/or expanding pre-existing capabilities at individual, organisational, and institutional levels to plan, conduct, and disseminate evidence-based knowledge [9]. The **individual level** includes researchers and research teams; the **organisational level** is composed of university research departments, think tanks and similar organisations; and the **institutional level** encompasses the regulatory environment and includes governmental bodies and policymakers [10].	
**Conflict and conflict-affected:** Conflict, as used here, refers to violent armed struggle between hostile groups, resulting in over 25 battle-related deaths per year [11]. We use conflict-affected in accordance to the World Bank’s definition to indicate areas that may not be bearing the brunt of violence, as it is the case in Lebanon, but still experience high levels of institutional and social fragility as a result of conflict, for example in the form of an influx of refugees or internally displaced populations [12].	
**Protracted Conflict:** Hostile interactions that extend over long periods with sporadic outbreaks of open warfare fluctuating in frequency and intensity [13].	
**MENA (Middle East and North Africa) Region:** Covers 24 countries, namely the 21 members of the Arab League (Algeria, Bahrain, Djibouti, Egypt, Iraq, Jordan, Kuwait, Lebanon, Libya, Mauritania, Morocco, Oman, Palestine, Qatar, Saudi Arabia, Somalia, Sudan, Syria, Tunisia, the United Arab Emirates and Yemen), as well as Iran, Israel and Turkey [14, 15].	
**East Mediterranean Region (EMR):** As indicated by WHO, it includes 19 members of the Arab League (Bahrain, Djibouti, Egypt, Iraq, Jordan, Kuwait, Lebanon, Libya, Mauritania, Morocco, Oman, Qatar, Saudi Arabia, Somalia, Sudan, Syria, Tunisia, the United Arab Emirates and Yemen), along with Afghanistan, Iran and Pakistan [16].	

These protests have significantly changed in intensity, spread and frequency since 2019 onwards but got fully subsided due to government measures to control the spread of the COVID-19 pandemic in February–May 2020. Although it is premature to predict the post lockdown trajectory across the MENA region, it is speculated that protests will regain momentum, exacerbated by severe economic and fiscal crises compounded further by the viral outbreak, as it is the case in Lebanon where violent protests erupted in late April 2020 in response to severe economic hardship [20, 21]. Such an endemic fragility results in a serious rise in health burdens and inequalities that place serious pressure on the region’s health systems which are already coping with the protracted nature of conflicts [22]. The MENA region thus needs continuous strengthening of its health research, at the institutional, organizational and individual levels to address these highly volatile health and social needs (See Table 1 for definition) [7].

In recent years, the MENA region has been one of many sites of increased investment and interventions in health research capacity strengthening. Programmes such as the EU-funded Research Capacity for Public Health in the Mediterranean (RESCAP-MED) [23], the UK-funded Research Capacity Building and Knowledge Generation (RECAP) [24] and Research for Health in Conflict Middle East and North Africa (R4HC-MENA) [25] projects are examples of recent and ongoing MENA-focused health research capacity strengthening interventions. R4HC-MENA, focused entirely on strengthening research capacity in the conflict-affected MENA region by implementing contextually relevant activities, is an interdisciplinary partnership led by King’s College London with UK partners such as Imperial College London and the University of Cambridge, MENA-based partners that include the American University of Beirut (Lebanon), Birzeit University (Palestine), Hacettepe University (Turkey) and King Hussein Cancer Centre (Jordan), and several humanitarian and policy organisations. Such activities include developing and delivering accredited multi-disciplinary courses, mentoring senior leadership at national and global/multilateral institution levels, and developing innovative Learning Technologies and Informatics platforms for distance learning [25].

Lebanon, which is the focus of this paper, is an upper middle income country in the Middle East with the highest refugee density worldwide following the Syrian crisis [26, 27]. It has been politically and socioeconomically influenced by regional conflict and crisis, as well as its own civil war (1975–1990), Israel’s Second Lebanon War (2006) and protracted internal strife (See Table 1 for definition). Although there has been a long-standing engagement with conflict and health research in Lebanon [28], research outputs in the country were negatively affected by instabilities in war times [29], but managed to quickly recover following the end of the civil war [30]. However, recent regional mobility of conflict-injured patients and the high influx of refugees have stimulated a renewed focus on conflict-related health research [31, 32]. Note that Lebanon has a long history of academic research compared to other MENA countries and has one of the highest numbers of publications and researchers per capita in MENA. It also has strong medical and health research provided mainly by university hospitals such as the American University of Beirut Medical Center and Hotel Dieu de France, both respectively affiliated to the large private and prestigious universities- the American University of Beirut (AUB) and Université St-Joseph (USJ). Lately other universities, including the Lebanese University, Balamand University and Beirut Arab University, are also focusing more on enhancing research capacity and outputs. However, most of Lebanon’s research production comes from the AUB which has made considerable efforts to promote research production and translation [33, 34].

**Fig. 1:**
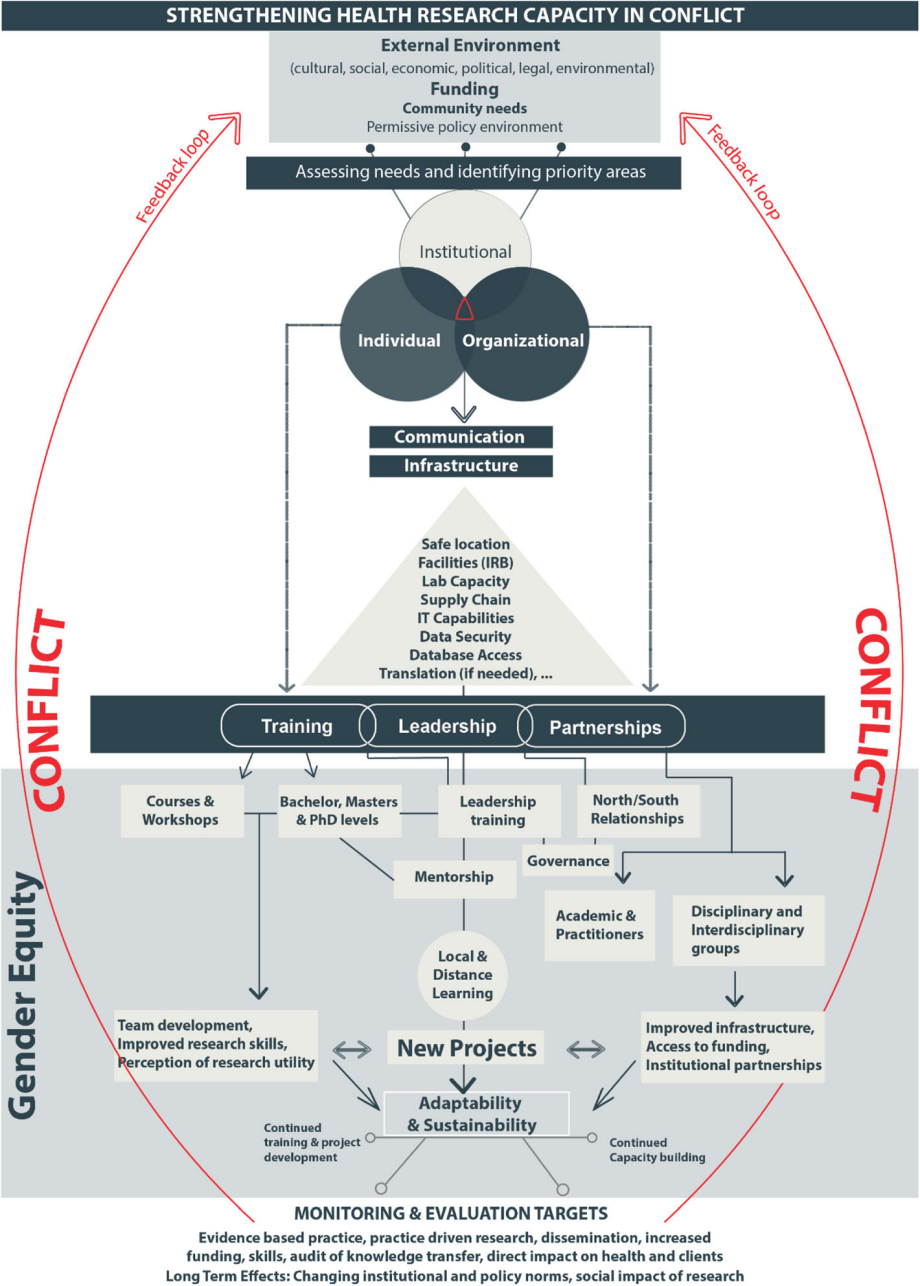
Conceptual framework for health research capacity strengthening in conflict [7]

An analysis of the country’s capacity for conflict-related health research is currently lacking, even as evidence for designing contextualized capacity interventions becomes more urgent. Building on a conceptual framework on capacity strengthening of health research in conflict-affected countries that focussed on the MENA region [7] (Fig. 1), we conducted a needs assessment in Lebanon to answer three main interrelated questions: What is the current capacity for conflict and health research in Lebanon? What are the challenges and gaps in conflict and health research in the country? And, lastly, what are the preferred tools and mechanisms of strengthening the country’s conflict and health research capacity from the perspectives of academics, humanitarian actors and public sector officials? The main aim of this paper is thus to analyse the strengths, weaknesses, opportunities and threats to conflict and health research capacity strengthening in Lebanon.

## Methods

We employed a qualitative approach using semi-structured interviews with academics, humanitarian workers and public sector officials as the main method of data collection. Due to scarcity of data sources on health research capacity in Lebanon, we opted for a qualitative approach that would additionally contribute to developing and refining interventions [35, 36]. In a previous related study, our research group designed a working conceptual framework on capacity strengthening of health research in conflict-affected settings, which was the basis for the topic guide for the semi-structured interviews [7].

### Recruitment and sampling

We used purposive sampling followed by snowballing sampling to identify interviewees. To achieve maximum variation, we sought out research participants from diverse geographic areas, ages, genders, and specialties (Table 2). Key informants consisted of three professional categories: 1) academics from various universities across Lebanon, 2) humanitarian workers working with local or international non-governmental organizations (NGOs) or United Nations (UN) agencies with operations in the health sector, and 3) public sector officials working at Ministry of Public Health (MoPH) and the Ministry of Social Affairs (MoSA). All research participants worked professionally in fields related to health, conflict and research in Lebanon. We included academics working at universities that provide postgraduate degrees as these tend to be more inclined towards research than universities that only provide undergraduate programmes. Out of the 39 informants approached, 9 refused to participate or did not reply to the invitation email and 2 subsequent reminders, leaving a total sample size of 30 research participants. We continued recruiting key informants until we reached thematic saturation.

**Table 2:** Characteristics of the key informants interviewed

Characteristics	Academics (***N*** = 18) n (%)	Humanitarian actors (***N*** = 8) n (%)	Public sector officials (***N*** = 4) n (%)
**Gender**			
Female	15 (83)	5 (62.5)	2 (50)
Male	3 (17)	3 (37.5)	2 (50)
**Nationality**			
Lebanese	17 (94)	5 (62.5)	4 (100)
Non-Lebanese	1 (6)	3 (37.5)	n.a
**Country of training**			
Lebanon	4 (22)	3 (37.5)	3 (75)
Europe	9 (50)	2 (25)	1 (25)
North America	4 (22)	3 (37.5)	n.a
Other	1 (6)	n.a	n.a
**Location in Lebanon**			
Beirut	15 (83)	4 (33)	3 (75)
North Lebanon	2 (11)	1 (17)	n.a
Mount Lebanon	1 (6)	n.a	1 (25)
Bekaa	n.a	1 (17)	n.a
Outside Lebanon^a^	n.a	2 (33)	n.a
**Highest degree obtained**			
PhD	16 (89)	1 (12.5)	1 (25)
MD	n.a	2 (25)	n.a
MA/ MSc/ MPH	2 (11)	5 (62.5)	1 (25)
BA/BSc	n.a	n.a	2 (50)

*n.a* Not applicable

^a^Canada

### Data collection

We conducted 30 face-to-face interviews between February and May 2019 across Lebanon. Semi-structured interviews were conducted in locations specified by the interviewees and were recorded when permitted to do so by the participant (*n* = 25, 83%), otherwise taking detailed notes of the interview (*n* = 5, 17%). The interviews were conducted in either English (*n* = 24, 80%) or Lebanese Arabic (*n* = 6, 20%). Most participants (*n* = 27, 90%) agreed to be quoted in anonymized form. Using a topic guide (Additional file 1), questions addressed experts’ perception of research in conflict settings, strengths and challenges in conducting research in Lebanon, and recommendations to strengthen conflict and health research capacities in Lebanon. To ensure clarity and relevance of questions, we consulted key experts from each professional category for the development of the topic guide. The interviews lasted approximately 45 min. Interviews were first transcribed into English and Arabic and transcripts in Arabic were then translated to English. All transcripts were anonymized using a unique identifier for each participant. The assigned code, used for citation purposes below, was composed of a letter that refers to the professional category of the participant [Academic (A), Humanitarian worker (NGO) and public sector official (M)] followed by a number that represents the chronological order of the interview. For example, A9 codes for the 9th academic and M2 for the second public sector official that we interviewed. We used NVivo 12® for data management and thematic analysis.

### Thematic analysis

We adopted the 6 phase process described by Braun and Clarke [37]. First, two investigators (NE, EK) worked independently on coding two transcripts line by line. Together, they discussed their coding approach to identify similarities and differences, thus avoiding interpretability bias, and created a thematic framework for data analysis. Second, the same investigators completed the open coding and started identifying emerging categories. Members of the research team (GHA, NE and EK) met on several occasions to reflect on the findings and identify the candidate themes and sub-themes. The latter was shared with other team members to reflect and finalize the results. Finally, a complete narrative of the findings was generated, supported with quotes from individual interviews. An early version of the study was also presented to the R4HC-MENA Executive Board meeting at Cambridge University in September 2019 and at the Global Syrian Refugee Crisis Conference in Gaziantep, Turkey, in October 2019. We then used the member checking approach to ensure confirmability and credibility. We used the consolidated criteria for reporting qualitative studies (COREQ): 32-item checklist for reporting the results.

### Increasing rigor

To further increase rigor, we focused on achieving both credibility and reflexivity. Regarding credibility, all discussions, except 2, were audio recorded, transcribed verbatim, accurately translated into English as necessary, and utilized as the main data repository. Once reaching data saturation, a decision was made by all team members to cease data collection. As for reflexivity, and to limit biases, all team members were involved in the analysis and interpretation of the results.

## Results

### Demographics

The 30 participants in this study were 18 academics, 8 humanitarian actors, and 4 public sector officials (Table 2). They are based in four different regions of Lebanon – Beirut, Mount Lebanon, north Lebanon and Bekaa – all of which are central areas where major NGOs, education and research institutes with a focus on health research are located. The 18 academics interviewed in this study collectively work at 7 out of a total of 11 accredited universities in Lebanon that provide health research training at the postgraduate level [38].

### Emerging themes

The 11 main themes that emerged are in accordance with those specified by the conceptual framework previously designed by our research team: perception of research capacity strengthening, research culture, current capacities and strategies of universities, research skills, infrastructure (data availability & ethics), funding & sustainability, partnerships (local & international) and the role of women working in Lebanon in health research [7].

#### Perception of research capacity strengthening

When asked about defining research capacity strengthening, the responses varied significantly ranging between considering it as a “training” mostly by local informants to a more detailed understanding targeted towards the individual, organizational and institutional levels. These variations were highly related to experience in previous capacity building interventions. Nonetheless, the term “capacity building” was used regardless of the context. For instance, locals used this term more often than those working with international NGOs who preferred to use the term capacity strengthening since *“building means starting from scratch which is rarely the case”* **(NGO2)**.

#### Research culture

Many informants pointed to a ‘lack of research culture’ as a major challenge for conducting health research in Lebanon:

“*I don't think we are in Lebanon so strong in terms of research, conducting research; we don't think about research as a major power”* **(A6)**

*“We [Lebanese] spend a lot of money on it [education]. But then when it comes to moving forward, making innovations, I don't think we are the culture that nurture this environment of research”* **(A12)**

Few informants also described hierarchical and challenging working conditions that can be counter-productive.

*“I might be very negative but I see a lot of motivated young researchers whose work is repressed by people at powerful positions … This is a major barrier to the progress of research”* **(A1)**

However, at the individual level, all of the informants expressed willingness to strengthen their health research capacities by making the best of the available resources. Career progression via promotion and improving job prospects were considered the main drivers for enrolling into trainings, courses, internships and volunteering opportunities; along with personal motivation.

*“I am not strong enough in qualitative research so when I knew about the course, I directly got involved despite that the university didn’t cover the expenses. I like to improve my skills. It is something I have deep inside.”***(A1)**

#### Health research strategies & capacities of universities in Lebanon

It was clear from the interviews conducted that there were huge disparities between the universities in Lebanon in terms of budget, qualifications, human resources and infrastructure. All of those interviewed agreed on the importance of research and considered it to be a major pillar of the universities’ strategies. Some universities, especially the highly ranked ones, allocate some of their budgets to fund research to complement external grant income. Changes at the organizational level and pushing academics to conduct more research are favoured to obtain accreditation from the Ministry of Higher Education, or to adhere with international standards of research and to gain a better reputation to attract students particularly for the private universities.

*“The university puts a lot of money in research, with a certain amount of the budget allocated specifically for this purpose”* **(A5)**

To incentivize academics to conduct research, most of the universities are considering publishing and research as major requirements for promotion and/or qualification for tenured positions.

*“So there's a promotion system. If you want to get promoted, you have to publish […] So there's a percentage. It's 50% research, 30% service and 20% teaching.”* **(A4)**

One of the informants highlighted that one of the leading universities is also adopting a strategy of forming research institutes and centres within the university, which focus only on providing executive trainings, courses, and knowledge to policy schemes along with knowledge production and research that could enhance the learning experience at the university.

*“along with education, we have multiple Interfaculty Research Centers and Institutes which deal with emerging health issues at the national and global levels.”* **(A7)**

However, there were complaints from academics in smaller universities about not having time to conduct research as they are overwhelmed with other duties so that they try to conduct research in their extra time.

*“If the department asks me to teach 5 courses every semester, I don’t think that I have much time left for research..”* (**A13)**

#### Research skills

According to most of the informants, individual research capacity for postgraduates is relatively high in some areas (epidemiology, health interventions, biostatistics) focusing on quantitative skills, especially in the top five universities in Lebanon. The research skills of undergraduates are considered by most of the respondents to be mostly introductory and insufficient for conducting rigorous research.

Nonetheless, all universities do provide courses on research and writing skills, but this tends to be at the postgraduate rather than the undergraduate level. Altering the content of courses would be challenging, as it would require new accreditation from the Ministry of Higher Education along with the internal institutional bureaucracy which is very time consuming. As a result, some informants admitted that they prefer to give extra-curricular workshops to cover key gaps in research skills at the undergraduate level.

*“They don't have this course (research design), I think they take a statistics course and introductory research course but they don't know what it means on the undergrad level… You cannot rely on undergrads to collect data if data needs interaction with participants… You need to get someone at the graduate level”* **(A9)**

*“So if we have a gap in our curriculum related to research or even to the theoretical courses we bring expert and we do workshops and conferences… we are trying to fill the gaps with some extracurricular activities”* (**A14)**

The gaps identified by most of the informants are in the social sciences, history, and political economy and mainly in qualitative research designs despite stating that mixed methods are important for a more holistic understanding of the health impacts of conflicts.

*“The other thing even on the postgraduate level, qualitative research is hardly taught. This is another main issue … we do quantitative blindly and the qualitative, which I think is much more significant for us … is not … I waited for my PhD to take a qualitative course. And still, I took a summer qualitative course when I was already a faculty to go over the themes...”* **(A9)**

*“…At times qualitative studies, which bring about a treasure of information that statistics do not often give …”* **(A10)**

When asking informants working in the humanitarian field, they emphasized that graduates from Lebanese universities have excellent quantitative research skills (depending on the university attended). However, they mentioned that there is a high demand in qualitative methods and tools of analysis to understand impact evaluations which fresh graduates lack.

*“We do a lot of interventions in the field, but we never understood the impact of the intervention and we don't know if the impact achieved was the result of what we did or result of other factors… So I think in order for us to replicate some interventions and to understand the weaknesses or the failure of some interventions, it's very important to understand the impact. We need qualitative research”* **(NGO3)**

#### Ethics in research

Interviews also indicated that monitoring of research quality and ethical approval in Lebanon are usually to meet the standards of international funders rather than local governments. Thus, ethics was a major theme highlighted by all informants. Most of the academics interviewed had Institutional Review Boards (IRB) at their institutions for granting approvals before conducting any research involving human subjects. The only downside of the IRB was the delay and difficulty in obtaining approvals especially when working with vulnerable populations. However, an informant mentioned that having an IRB is not enough to ensure ethical research.

*“All kinds of research with the proviso that the culture, context and participants full rights are respected… and that they ‘benefit’ from the research one way or the other”* **(A10)**

An informant also mentioned the wellbeing of researchers, which is mostly ignored, as a key ethical determinant that should also be considered along with that of participants.

*“At times qualitative studies can be a traumatizing experiences. Thus, it is important to have good solid ethical review for the research and psychological support to both researchers and participants when needed.”* **(A12)**

As for NGOs, there is a huge difference between international and local humanitarian actors. For the former, there are strict rules when it comes to dealing with vulnerable populations, whereas for the latter, this concept might be lacking. Due to the limited regulation of research in the field, there are serious concerns about researching vulnerable populations, with infringement of the international regulations of ethical research especially in conflict settings.

*“Our main mandate is protection and protection of cases. Confidentiality is for example one of the 0 tolerance for [Organization name]. We also have ethics guidelines where everyone, every student needs to read, sign and abide. It’s very strict, it’s 0 tolerance.”* **(NGO1)**

The absence of oversight on research ethics in Lebanon was a recurrent concern. For some, governmental oversight on research ethics would be detrimental, for fear of it being restrictive, especially when ‘sensitive’ topics are researched. For others, the repetitiveness of the same research projects on the same populations (for example, Syrian refugees) is detrimental, often being intrusive, dehumanizing, or inappropriate (an example given is asking people who are hungry to document their meals). As indicated by a public servant*,*

*“Usually NGOs working in the field are not affiliated to any IRBs in hospitals or universities.[…]. I'm sure there's a lot of research at the being conducting on Syrian refugees. And unfortunately NGOs working in the field are not aware of these requirements of having ethical approvals, of the informed consent process, and all of these. The research that I come countered with by accident because we have no mechanism to find out what's going on. The way we catch them is when they take biological samples and they want to export it outside the country and they need the approval of MoPH. There is a study that we ended up putting under control … it was done between Lebanon and a university outside .. it was a DNA testing. They considered it not a big deal since they collect saliva and cells with toothpicks. I forced them to get ethical approval and to understand what they're doing with the samples beside research.”* **(M2)**

#### Access to data

As indicated by **M3** “*we (local centres of MoSA) don’t conduct research but provide data for institutes following the permission from MoSA*”, health research data if available can be provided by MoPH and MoSA. Private hospitals also have their own databases that can be used in research. However, as indicated by most academics, access to data is a huge challenge that limits the ability to conduct impactful research.

“*There is no databases so you know if you want to have information about the patient, about any health problem, it's really hard to get what you need to know about it.*” **(A4)**

*“So when you go to private hospitals, they have a different interests. if there is a conflict with business because they don't want to have their image tarnished because of certain quality indicators which are not optimal or because that will reduce the number of patients coming to their services, they're very resistant to conduct research. And if they do conduct research, they show only the positive side of it.”* **(A6)**

Interviews with humanitarian agencies indicated that data collected is for basic analyses to inform the programmatic direction of the agencies, and not necessarily for statistically advanced quantitative or in-depth qualitative studies and thus can be of minimal use to academic research.

Moreover, one informant highlighted the problems that arises from interpretation of available data especially in conflict-affected settings; *“Data though can be misleading depending on how it is interpreted and by whom.”* **(A10)**

#### Partnerships

All academics interviewed thought that partnerships and collaborations at both the national and international levels were crucial and highly beneficial for the progress of work. They suggested that these partnerships could help to mitigate the gaps and barriers of health research in the country in terms of infrastructure such as expensive equipment. For instance, international partners provide an opportunity for the local academic institutes to get access to facilities and equipment which are unavailable in Lebanon and/or are too expensive to purchase and maintain. Similarly, several local universities have joint PhD programs with international universities for local students to produce high quality research which is context-oriented while still getting access to facilities available in international academic institutions.

Informants also mentioned examples of exchange of faculty members between universities as a way of knowledge and strengthening experience for both faculty and students in both institutes.

Partnerships were also considered as an opportunity to bring grants to the local institutes to conduct health research as it is more appealing for international funders to fund consortia rather than a single university or institute in Lebanon. Informants mentioned projects funded by the European Union, UK Research Councils, and National Council for Scientific Research (CNRS) included partnering with other local and international universities.

Partnerships between academics and local NGOs were considered to be important as they provide access to refugee camps and local communities and help with logistical arrangements and security of data collectors.

*“As an academic institution […] you need to collaborate with an NGO for them to provide access to camps […] it's also always better to have someone from the NGO to stay with you for security measures or if anything is to happen on the field as researcher”* **(A8)**

The MoPH and MoSA informants also highlighted the importance of collaborating with UN agencies such as WHO, United Nations Educational, Scientific and Cultural Organization (UNESCO), United Nations Development Programme (UNDP), United Nations International Children’s Emergency Fund (UNICEF), United Nations High Commissioner for Refugees (UNHCR) … and other international NGOs that sponsor and deliver capacity strengthening workshops, trainings and seminars for the public sector officials working in these ministries.

*“Usually when there is a training that the ministry wants to conduct, we approach these organizations for two things: first for providing technical experts to conduct the trainings, because it provides a better credibility for the training, and second for logistic support… but these trainings are not for research skills”* **(M2)**

Some of the topics that were mentioned by the informants include: *“psychological support, service provision, finances, management, Training of Trainers (ToT), Communication for Development (C4D)… following the Syrian Crisis, more trainings were conducted on topics related to child protection and gender-based violence… the content of these trainings is regularly updated.”* **(M1)**

#### Cross sectoral knowledge transfer

Despite highlighting the importance of partnerships with local and international actors, it was found that most of these partnerships were within the same category: academics-academics, or ministry with NGOs.

The lack of coordination between NGOs for example, out of competition or lack of communication in a highly unregulated field was reported to be a major drawback.

*“I feel there is no universal coordination at least per country and the national level, to divide efforts in a way that's equitable on all levels so there could be too many interventions* *happening on one topic, let's say reproductive sexual health, just an example. And too little happening on mental health. So if efforts were combined and property divided, I feel it would be more beneficial.”* **(A8)**

Even within academia, an informant mentioned that the current ‘academic silos’ coupled with the “fragmentation of academic disciplines” and ‘over-specialization’ of research areas is a major limitation for interdisciplinary research in Lebanon.

On the other side, most of the informants considered that policymaking is not evidence-based, relying on what are called ‘cold’ experts (the opinions of ministers, public personalities, funders, etc.) rather than empirical research.

However, an informant from MoPH blamed this lack of coordination on the academic institutes that “live in their own bubble” and disseminate their findings in publications that policy makers have no time to read. The informant highlighted the importance of involving policymakers in a research from the early stages to improve the collaboration.

*“For example will we (MoPH) know about the academic research when it is done? Its recommendations? There is no link... They present their findings in conferences and then say policymakers are not responsive .. How would we know about the research, we don’t have time… Policymakers should feel that they are setting research agenda and are more involved in the research process”* **(M2)**

#### Funding & sustainability

For many, the main challenge is not the lack of individual research capacities, especially when it comes to quantitative research skills, but more the infrastructural capacities (database) and the availability of research funding. Funding and sustainability have been identified as two of the main interlinked challenges for capacity strengthening for conflict and health research. Informants argued that sustainable capacity strengthening is largely determined by the sustainability of funding. For laboratory sciences specifically, building of individual capacity in microbiology, for example, is limited when basic research infrastructure and sustainable funding for technologically and labour-intensive laboratory-based research is lacking. At the core of this in Lebanon is the near absence of local funding, and the high dependency on highly competitive international grants and schemes. Researchers describe competing for rigid international grants and research funding, while working in highly unstable research settings, require a high level of flexibility and adaptability. Most institutions struggle to pay competitive salaries for pharmacists or medical doctors to conduct and generate evidence-based research.

Most of the informants indicated that adapting research questions and methods to secure funding was fairly common. Such a high level of instability is also a disincentive for long-term planning.

*“Naturally that's what happen most of the time… We have to go work with whatever the market is.”* **(A2)**

However, few informants mentioned that funding is not an issue if a well-designed project is prepared especially for internal funding provided by universities or by local industries.

*“if we prepare a decent research protocol. I think that we can get funds.. Even the scientific associations, if there is a good protocol... Money is not an issue”* **(A1)**

*“My motto is if you have the will you have a way…I think the other factors become secondary…. So funding could be a factor, but again it's not THE major barrier or challenge.”* **(A6)**

As for the humanitarian sector, according to informants, funding for humanitarian relief and operational research has slowly decreased, potentially leading to increased competition among local NGOs that would limit even more the coordination among these humanitarian actors. As for international NGOs, fewer resources are allocated to them in Lebanon and thus they are currently withdrawing slowly.

#### Women in health research

When asked about women’s involvement in health research in Lebanon, most of the informants, regardless of their gender, indicated that discrimination is not a major issue as women are capable of reaching high positions in academia.

*“Representation of women at our institute is Fantastic, more than 50%; 55% of researchers and teachers are women, 6 out of 13 deans are women and 2 out of 5 are vice presidents”* **(A5)**

#### Conflict: a challenge or an opportunity?

Most of the respondents were reluctant to consider Lebanon as a conflict setting as it is not struggling with ongoing armed clashes. But they considered Lebanon as a conflict-affected setting due to the huge influx of Syrian refugees. At the research and development level, most of the informants agreed that being a conflict-affected country has led to an increase in research funding and opportunities. However, most of the research funded was focusing primarily on refugees.

*“I guess what happened is that many people started doing research related to the crisis because there are opportunities or there is money with this coming in… It has brought some opportunities like the Syria Lancet Commission, which has been great for AUB…”***(A7)**

Host communities were also considered in both health research and humanitarian interventions which made them benefit from getting access to services that were not prioritized before the Syrian crisis, especially services for child protection and those that address gender-based violence.

Despite providing research opportunities and employment for locals researchers who obtained field experience, an informant highlighted that locals “*learned by doing, by being exposed, rather than learning principles of intervention in an academic normal pace setting*” **(NGO5)**, which due to the lack of local expertise led to “*humanitarian efforts in all of the conflict affected settings in MENA to be led by international non-Arab experts who do not necessarily understand the context*” **(NGO5)** which might lead to inconsistent interpretations.

Another factor that was raised by most informants but not considered in the topic guide, was that the basic medical training provided in Lebanese universities, does not include treatment of war injuries, trauma, health provision in conflict settings, or health research in such settings. Public health professionals are able to conduct research in conflict settings but most medical doctors have limited research skills needed for such a context.

*“Even people in medicine are not trained in research through all of medical school”* **(A9)**

“*Especially in the war zone to begin with. Don't forget it’s a totally new pathology for most surgeons.”* **(NGO4)**

#### Recommendations

Clear recommendations regarding more effective health research capacity strengthening interventions that target the individual, organizational and institutional levels were indicated by many informants.

Regarding better regulation of research, particularly research ethics; workshops on ethical research especially for local NGOs were recommended.

To address limited coordination between academic bodies and ministries to provide evidence-based policies, roundtables and working groups to discuss priorities of health research with the different stakeholders including policy makers, local NGOs, International NGOs and academics were thought to be a top priority going forward.

*“It is important to have a research group to work on setting priorities in order not to have irrelevant research. The results should be then summarized in a policy brief a maximum of 4 pages with direct and simple recommendations to send to policy makers.”* **(M2)**

In order to have more sustainable funding schemes, it was recommended by most of the informants for research groups to be involved in multidisciplinary and context-specific consortia or research networks like the Reproductive Health Working Group and the Lancet Palestinian Health Alliance [39]. Those networks would also provide the opportunity of capacity strengthening of researchers via training and mentorship as they go to other countries with advanced technologies to be trained with the latest research developments.

Capacity strengthening activities for the humanitarian actors and graduates with medical and health backgrounds need to focus on basic qualitative and advanced qualitative and quantitative methods. An informant also added that qualitative research especially going beyond focus groups and interviews and into ‘cutting-edge’ tools is needed to benefit both communities and researchers (the example given was story-telling as a research method that may also have benefits to research participants). When asked about the type of training to be provided, most informants suggested that accredited certificates in health research methods are highly desirable for all as an incentive for participation. Similarly, introducing a module about medical practice in conflict that addresses blast injuries, trauma, and medical practice under severe conditions was also recommended and considered to be an urgent need given the geopolitics of the MENA region.

When asked about teaching modalities of the certificate, most of the informants highlighted the importance of e-learning which has less burden on both financial and human resources and would be highly appealing especially if it is accredited. However, most agreed on having a blended learning course, rather than purely e-learning, which might not provide the most effective learning experience especially to future fieldworkers. An informant also mentioned the possibility of having training that is fully distant but with innovative tools that make the courses more interactive.

*“I think, it opens doors to expertise that maybe we don't have that you can get from other universities…. I think it is great when you're talking on the theoretical level… but to go from there to design a research study that is a big step …. it saves time, money and human powers and then we can focus the energy on things that need this human interaction.”* **(A2)**

## Discussion

In this study, we have provided an overview of capacity needs for health research on conflict in Lebanon based on strengths and challenges at individual, organizational and institutional levels which are summarized in Table 3. However as these levels are strongly interconnected, any strength or weakness in one of these levels will directly impact the other two levels [9]. Most of the findings are consistent with the international literature on research capacity strengthening in Low and Middle Income Countries (LMICs) including those affected by armed conflict and political unrest [40, 41]. The key themes emerging from this work are categorized below as strengths and challenges of health research which would support the design of impactful research capacity strengthening interventions.

**Table 3:** Strengths and challenges of conflict and health research in Lebanon at the individual, organizational and institutional levels

Strengths of conflict and health research in Lebanon	
Level	Element
**Individual**	Most faculty have research training from US and European countries with relatively few PhD programs in Lebanon
	Open and willing to learn new ways of teaching new content
	Aware of own limitations with teaching research
	Qualitative and quantitative research is valued by researchers, with bias toward quantitative
	Experience with international collaborations and are comfortable with these
	Strong interest across all junior researchers in developing research skills
	Highly receptive to online learning teaching methods
	Consider that continuing education with accreditation can provide the necessary skills while working
**Organizational**	Lebanon is considered a major health research hub in the region
	Civil society interested in developing research critique skills, especially qualitative
	Research is a part of each university’s mission
	Some universities like AUB and USJ are considered regional hubs for health research
	There is an individual IRB mechanism for most universities and hospitals
	Increased collaborations between universities, NGOs, and Ministries
	Serious attempts to fill gaps in the curricula in research skills and rising health topics
	Capacity strengthening for health research in conflict is prioritized internationally
**Challenges of conflict and health research in Lebanon**	
**Individual**	Faculty members with experience in designing and conducting qualitative research studies is limited
	Commitment to e-learning based certificates
**Individual/Organizational**	Limited mentorship opportunities within most universities
**Organizational**	Huge disparities in human resources and research infrastructure among universities and among local NGOs
	Teaching methods and topics vary widely
	Power dynamics within certain universities negatively impacts research
	Concentration of research in few universities such as AUB, USJ and LU.
	Complicated university bureaucracy
**Organizational /Institutional**	Lack of sustainable funding
	Gap between research conducted, policy and practice
	Limited communication with different stakeholders
**Institutional**	Limited access to data
	Political instability
	Data quality of national datasets
	IRB for some studies is decentralized and unregulated
**All levels**	Lack of research culture in Lebanon and the wider MENA region
	Questionable ethical standards in research especially for local NGOs

One could argue that these strengths and challenges impact research in general and are not limited to health research, with few exceptions including qualitative research designs for health research topics, ethical conduct of health research, and preparedness to practice medicine in conflict settings. Therefore, using the same approach, suggested in this study could also improve research in conflict-affected countries at the individual, organizational and institutional levels. However, further research is required to validate this assumption, especially as strengthening research requires more financial and human resources. This would result in additional challenges and constraints where the suggested approaches herein might prove invalid especially that the inclination to health research in low-resource humanitarian-oriented conflict settings might not be applicable to other types of research or for research as a whole.

### Key strengths

The needs assessment highlighted several enablers within the Lebanese context that can contribute towards impactful capacity strengthening interventions. For instance, both researchers (especially early career researchers) and institutions are incentivized to improve their research capacities and outcomes for various reasons. The former is driven by the need to increase their job prospects in a highly competitive job market and the latter to improve their reputation as centers of research given that there are 47 universities in Lebanon, one public university hosting 79,360 (38%) students and 46 private for-profit organizations hosting 131,360 (62%) students that mostly rely on student fees [38, 42, 43]. Therefore, despite being a relatively small country in terms of size and population, Lebanon has recently been ranked as the third in the East Mediterranean Region (EMR), and MENA, after Kuwait and Tunisia in terms of the number of publications on biomedical and health research per capita [34]. It is also important to note that there are huge disparities among the universities in Lebanon with some being considered as centers of research excellence within the region by contributing most significantly to the country’s research outputs and to setting considerable momentum to continuously improving their research capacities. For instance, most of the research outputs originate from the Lebanese University, USJ and AUB with the latter contributing the most and is being ranked the 10th in biomedical and health research output in EMR [34, 44, 45].

Lately, the Lebanese highly privatized educational system has shown resilience to adversity, at least in the short term. Following the country’s lockdown to control the spread of COVID-19, academic institutes have shifted to online learning. Vast efforts are underway to provide innovative distant learning approaches that could provide similar levels of academic experience as in face-to-face learning modalities [46, 47]. The current attempts to create a reliable online learning platform is an opportunity that can be used for a capacity strengthening intervention to be more inclusive to include those residing in ongoing armed conflicts [48, 49].

Another opportunity which universities are taking advantage of is the increasing trend of partnerships between local and international academic institutions which increases bidirectional knowledge sharing. For instance the country, along with Jordan, Morocco and Tunisia, has the highest levels of co-authorship in MENA [33]. Partnership has already been considered a pillar in capacity strengthening of health research in other settings as it supports collaborative, multidisciplinary and multi-sectoral work which in theory is aimed at addressing power imbalances and inequities [50–53].

Although Lebanon is ranked the 145th out of 153 for the global gender gap index [54], it was interesting to learn that almost all informants, regardless of gender, indicate that the health sector, including research, provides a permissive environment for female researchers to work and advance in their careers (as several Deans and Vice Chancellors of Research are women). However, health and education are traditionally considered female- friendly compared to other Sciences Technology Engineering & Medicine (STEM) disciplines, so the results reflected in this study cannot be generalized or considered on a larger scale. Indeed a recent report on women in academia in the Arab world found that women lead fewer than 7% of higher-education institutions [55]. Thus more research needs to be done on this topic especially that another recent study highlighted a high level of exploitation and alienation of research assistants, who are mostly women, that are working on UK funded research projects focusing on Syrian refugees in Lebanon. The study emphasized on how the hierarchy in academia, which was also mentioned by few of our informants, could reinforce cultural and academic inequalities including gender [56]. Despite of the variation in the findings and building on the informants’ responses, this factor can be considered as an opportunity for both the health research field and the cause of Lebanese women in general as having more women in the field in leadership positions will put women’s issues at the front of the health research agenda and advocate for more inclusive and diverse research [57].

### Key challenges

Despite the opportunities and strengths in terms of human capital, research capacities of certain universities, and the perception of improvement within academia, there are several challenges, besides political and economic instability, that impact health research in Lebanon.

A factor which was highly expected was the lack of research culture at the country level which limits the impact of any health research despite its importance and relevance to local challenges. Unfortunately, this is an issue which the whole region is struggling with as only 1.6% of the global biomedical research output originate from the region [34]. Research culture could be improved and developed by joint efforts between policy makers and the institutes of higher education to provide opportunities for research grants, local and international collaborations, cross-sectoral knowledge transfer, and skill acquisition [58–60]. It can also be promoted at the community level by publicizing research through the media including social media [61, 62].

The issue of research culture leads to the other challenges of health research and its strengthening in Lebanon. For instance, there is a problem in cross-sectoral knowledge transfer in EMR, including Lebanon, where there is a lack of both demand for and supply of relevant research which could be attributed to political forces, political sensitivity of findings, limited funding and limited opportunities for interaction during the policy-making process [63–65]. Closing this gap, by engaging policy-makers in setting research agendas to meet their needs, is a key challenge for the health research system where even the best studies are rarely translated into action [66–68]. In Lebanon, there are a few centers that directly engage with policy makers to advocate for evidence-based policies like the Lebanese Center for Policy Studies, Issam Fares Institute-AUB, Global Health Institute (GHI)-AUB and Knowledge to Policy center (K2P)-AUB with the last two focusing primarily on health. These centers have been greatly involved lately in issuing policy briefs on political, economic and health system situation in Lebanon following the October 2019 uprising and the COVID-19 outbreak [69–71]. The Chronic Care Center (CCC)’s β -Thalassemia prevention program that was launched in 1994, in collaboration with MoSA and MoPH, is one of the few examples highlighting the benefits of direct collaboration between policy makers and health and research centres [72]. Key recent evidence use for policy in the country include passing of tobacco control law 174 and introducing a reporting mechanism for occupational violence as part of the 2019 accreditation criteria of private Lebanese hospitals [73, 74]. However, given that in crisis zones four systems – political, health, international humanitarian aid and health research – are involved in advocating for evidence use, there is a need for further national level action by engaging multiple stakeholders to set research priorities [75]. Achieving efficient multiple stakeholder coordination will also contribute positively to generating and sharing reliable data across the four systems and with providing additional funding opportunities which would mitigate both of these constraints to health research in Lebanon.

With regards to funding, according to the CNRS, the country allocated only 0.2% of its Gross Domestic Product (GDP) for research and development in 2007 [33]. As a result, universities in the country rely primarily on international funding schemes to support their research activities along with allocating part of their budgets for this purpose. The amount of money allocated varies remarkably among the universities in Lebanon with AUB being the lead, followed by the Lebanese University and USJ [45]. It is due to external funding that the universities in Lebanon are focusing on conflict and health research as it is an agency research priority [56]. Given the current drastic economic situation of the country, it is expected that universities will depend even more on international funding resources as they might not be able to allocate the same percentages of their budgets to fund research given that the number of registered students in private universities will severely decline. Thus, the issue of sustainable funding of health research remains unresolved until the Lebanese government is capable of adopting a research strategy that supports health research at the national scale.

Another serious issue, ethics in research, which was highlighted in this study is also linked to cultural factors. In the MENA region, and Lebanon, there is a strong collective orientation where the community plays a significant role in an individual’s life; resultantly achieving informational and decisional privacy of research participants could be challenging [76]. Obtaining consent, in its Western notion, from populations with multiple vulnerabilities such as Syrian refugees is also problematic given the coercive societal dynamics and context. So despite a thorough IRB process, conducting refugee research in practice remains ethically challenging [77]. Nonetheless, Lebanon lacks a unified system of research governance, and hence research regulation is greatly influenced by the policies of individual institutions and their IRBs [78, 79]. Obtaining unified guidelines of ethical research in Lebanon faces multifaceted complications so a direct and short-term mitigation plan would be to train fieldworkers working with local NGOs to understand better the concepts of privacy, confidentiality and consent while still adhering to the Lebanese context.

Interdisciplinary research is crucial in understanding and resolving complex public health problems especially in conflict [39, 80]. Qualitative and quantitative research methods are required to provide an enhanced understanding of the causes and consequences of conflicts while emphasizing personal experiences and narratives of those struggling from these conflicts is important [81, 82]. However, interdisciplinarity is not a strength in health research in Lebanon despite having few funding opportunities for such initiatives [83, 84]. Human and social science research is significantly ignored as a research study indicated that only 5% of CNRS and 9% of AUB research support goes to this field [33]. Qualitative research skills, at both undergraduate and postgraduate levels, appears to be a central weakness in the Lebanese educational system. In addition, students with social sciences background also struggle with quantitative research skills. Such weaknesses vary significantly depending on the university’s teaching and research capacity which, as highlighted earlier, vary tremendously. The concentration of research in few private universities, with relatively high tuition fees, limits the opportunity of graduates from other universities, and from more diverse socio-economic backgrounds, to be trained in conducting conflict and health research of high quality. This heterogeneity in health research training can be mitigated if graduates, regardless of their alma mater, are provided with extra-curricular/ stand-alone courses on interdisciplinary research methods in conflict that are suitable for a wide range of participants from different academic backgrounds.

While the initial focus was mainly on health research in conflict, this study also exposed a new fragility within the medical curriculum in the country. Medical graduates are not prepared to practice in conflict settings despite the fact that the region, including Lebanon, is highly vulnerable to instability [85]. Taking into consideration that any changes in the medical curricula across Lebanon would be challenging due to logistics and difficulties in reobtaining accreditation from the Ministry of Higher Education, specific postgraduate training could fill this gap by introducing training that focuses on medical practice in conflict settings. This would be taken by individuals interested in practicing in conflict-affected humanitarian settings across the MENA. The International Committee of the Red Cross (ICRC) has war surgery courses which could benefit local practitioners [85]; however, these courses are limited in scope as they focus exclusively on medical students at the Lebanese University.

Despite this study’s focus on Lebanon, the challenges and strengths of the country’s health research capacities presented herein are in accordance with similar studies that focus on health research in the MENA/EMR region especially in conflict-affected settings. The recent work of AlKhaldi et al. that focuses on research capacity strengthening in Palestine, also revealed that health research capacities in the country are relatively weak despite the recent increase in health research production. Guidelines for Health Research Quality and Standardization are not adhered to due to a lack of policies and resources, and Health Research Knowledge Transfer is a major challenge that requires contextualization and adequate implementation [41]. Regarding research skills, a needs assessment focusing on the West Bank, Palestine, published by R4HC-MENA colleagues, found that most of the respondents considered that they have a significant gap in their abilities and understanding of coding and analysis of qualitative data, and planning and designing research [86]. This is also supported by our study’s findings in terms of the need to focus more on qualitative research designs in Lebanon. Moreover, a recent bibliometric study focusing on research activity on non-communicable diseases (NCDs) in the MENA found that there has been an increase in research activity in the region that is heterogenous with respect to wealth, population size and type of NCD but with no clear correlation to the corresponding disease burden. The study suggested that international collaborations can support overcoming problems such as a lack of suitably trained researchers, low political commitment, and poor financial support; all of which have been indicated in our study [87]. Similarly, the paucity in health research and training which are contextualized to conflict settings is also a wider MENA region problem as “Emergency Preparedness” is one of the least studied fields in the EMR/MENA regions- 1.5% journal papers published in the field of public health research between 1995 and 2014 [88].

## Limitations

One of the main limitations is perhaps that the findings may not be generalizable outside the Lebanese context, although a follow-up comparative study focusing on MENA would enable broader interpretations of the key research objectives. However, the high level of consistency from the key informant interviews of the same participant type and the similarity between the findings of this work to those of quantitative studies focusing on health research in the MENA provided meaningful context. Another limitation is that most of the refusals or unanswered invitations were from the public sector informants. Therefore, there might be other themes that would be more relevant to the public sector that this study did not reflect due to the low number of participants from the public sector. However, those interviewed provided similar feedback to that of the other categories. Promoting inter-sectoral communication, which is one of the major recommendations of this study, would mitigate this problem in future research as more informants from the public sector will be more willing to participate in research activities.

## Conclusions

In summary, the study revealed that health research in Lebanon is characterized by considerable strengths in terms of human capital and research capacities of certain universities. However, there was considerable concern regarding research ethics among most informants, and workshops on ethical research especially for local NGOs were recommended. Guaranteeing sustainable funding of conflict and health research which was highlighted as a major challenge is problematic given the current economic crisis of the country and its lack of research strategy at the national level. Thus strengthening partnerships with international collaborators is the only way to secure funding at least in the short run. The impact of research on policymaking was also another recurring theme where organizing roundtables to discuss priorities of health research with the different stakeholders was suggested as a way forward.

For strengthening research capacity at the individual level, a great amount of interest around research methodology, conceptualizing and design was expressed. Moreover, providing knowledge about medical practice and dealing with war injuries was also considered as a need to fill the gap in the medical training provided at universities that do not currently focus on this aspect in medical training. Accredited certificates were considered as an incentive for participation and a combination of e-learning and mentorship was identified as having the most potential impact. These findings will be useful for guiding research capacity strengthening approaches within large international projects that will include developing academic courses, engaging with NGOs and policymakers to improve capacities for conducting useful research, and delivering trainings on health research skills in conflict settings. The study herein, supported by qualitative research methods, provides comprehensive insight and complements the findings of several quantitative bibliometric analyses of health and biomedical research in Lebanon and the wider MENA region [30, 34, 64, 87].

## Supplementary information

**Additional file 1.** Topic Guide.

## Data Availability

The datasets generated and analysed during the current study are not publicly available due to them containing information that could compromise research participant privacy/consent but are available from the corresponding author on reasonable request.

## Abbreviations

AUB: American University of Beirut
C4D: Communication for Development
COVID-19: Coronavirus Disease 2019
EMR: East Mediterranean Region
GHI-AUB: Global Health Institute-American university of Beirut
GNI: Gross national income
ICRC: International Committee of the Red Cross
IRB: Institutional Review Board
K2P: Knowledge to Policy Center
LMIC: Low and Middle-Income Country
MENA: Middle East and North Africa
MoPH: Ministry of Public Health
MoSA: Ministry of Social Affairs
CNRS: National Council for Scientific Research
NGO: Non-Governmental Organization
RECAP: Research capacity strengthening and knowledge generation to support preparedness and response to humanitarian crises and epidemics
NCDs: Non-communicable diseases
REDCAP-MED: Research Capacity for Public Health in the Mediterranean
R4HC-MENA: Research for Health in Conflict MENA
STEM: Sciences Technology Engineering & Medicine
ToT: Training of Trainers
UN: United Nations
UNDP: United Nations Development Programme
UNESCO: United Nations Educational, Scientific and Cultural Organization
UNHCR: United Nations High Commissioner for Refugees
UNICEF: United Nations International Children’s Emergency Fund
USJ: Université St-Joseph
WHO: World Health Organization
