# Supplementary material for: Assessing the capacity for conflict and health research in Lebanon: a qualitative study
Source: Confl Health. 2020 Aug 18;14:59. doi: 10.1186/s13031-020-00304-x (PMC7432458; doi:10.1186/s13031-020-00304-x)
Supplement: Supplementary file 1 — Additional file 1. Topic Guide. [file 13031_2020_304_MOESM1_ESM.docx]

**Additional file 1: Topic Guide**

**Conflict Medicine and Refugee Health Programs, Global Health Institute, American University of Beirut**

**Project:** Research for Health in Conflict in the Middle East and North Africa (R4HC-MENA): Assessing the Needs for Capacity Building of Research on Health in Conflict

**Questionnaire 1: Health & Conflict Workstream**

*Thank you for agreeing to be interviewed for this study. I will start with some broad questions around your own research / teaching / work as well as on research in conflict settings, before moving on to your own experience with research capacity building (through your teaching / work). Finally, I’ll move on to some questions around barriers and enablers for research capacity building. The interview will take no longer than 55-60 minutes. Before we begin, do you have any questions?*

**Questions**

1. **Could you tell me a bit about how your research / work relates to the topic of conflict and health / humanitarianism / conducting research in challenging environments / research capacity building?**
2. **What role does or should research play during conflict?**
   1. What aspects of research would be important during a conflict? Give examples
   2. What research gaps exist in settings of conflict (e.g. Lebanon)?
   3. Shaping policy and practice? Affecting the trajectory of conflict?
   4. Who determines the research agenda in your institution?
3. **What is your understanding of what research capacity building (for health) is or entails?**
   1. Probe on different levels: individual, organisational, institutional.
   2. Probe on whether informant has taken part in any training.

**Academics:**

*Thanks. Now, I’d like to move on to asking you some questions about your own experience with research capacity building through your teaching. These are a little bit more specific.*

1. **Are you involved in designing a course that involves research capacity building for conflict and health, or that is broadly relevant? If so, do you mind telling me a bit about the details of the course, in terms of how you incorporate research capacity building, as well as the logistics of the course - for example its participants (in terms of professional and academic background, etc)** [Skip to 7. if answer is *no*].
   1. Probe on teaching methods:
      1. Writing component
      2. Research methods
      3. Which disciplines research methods reflect
      4. E-learning
   2. Probe on logistics:
      1. How long is the course?
      2. How is it accredited?
      3. How much does it cost (also in terms of human resources required to deliver the course)?
      4. What funding options are available for participants?
      5. How do you attract students to your course?
2. **Thinking now about support and engagement beyond teaching on your course, are there any opportunities for students – such as institutional links, mentorship, support for publishing, or networking opportunities?**
   1. Are these useful?
   2. Do students go on to publish? What challenges do you experience when publishing outputs from course participants?
3. **Do you experience any challenges when incorporating research capacity building into your courses on conflict and/or health?**
   1. Are there any external political constraints influencing the content of your course or the undertaking of research by participants?
4. **How would you integrate a research capacity building component to your course?**
   1. Is there any specific infrastructure that would help you do this?

**Practitioners:**

*Thank you. Now I want to ask you a bit more about your role as a ‘practitioner’ and how you view research capacity building.*

1. **How do you view the importance of research in relation to your work?**
   1. Does research play a role in your work? How?
2. **In what ways can the research capacity of those working in humanitarian settings be improved?**
   1. Have you participated in any research training? Was it effective?

**All participants:**

*Thanks, that’s all really helpful. Now I would like to move on to some broader questions around research capacity building*

1. **Thinking beyond your own courses or work, what are the greatest challenges to research capacity building (for health) in conflict-affected areas?**
   1. Probe on:
      1. Political or structural dynamics
      2. Gender
      3. North-South relationships
      4. Relationship / proximity to funders
      5. Different levels (individual, organisational, institutional)
2. **Are there examples of good practice you can think of that demonstrate effective research capacity building within conflict and health?**
   1. What makes these successful?
   2. Probe on individual, organisational, and institutional level.
   3. Probe on sustainability.
   4. (If not asked previously) Do you think e-learning technologies can be useful for building research capacity for conflict & health?
3. **Are there any ethical issues that particularly pertain to research in complex environments?**
4. **Is there anything I haven’t asked you that you think is relevant to the issue of research capacity building in conflict-affected areas?**

*Thank you very much for your time and cooperation.*

*End of interview.*
